# Acoustic Energy Harvesting and Sensing via Electrospun PVDF Nanofiber Membrane

**DOI:** 10.3390/s20113111

**Published:** 2020-05-31

**Authors:** Nader Shehata, Ahmed H. Hassanin, Eman Elnabawy, Remya Nair, Sameer A. Bhat, Ishac Kandas

**Affiliations:** 1 Department of Engineering Mathematics and Physics, Faculty of Engineering, Alexandria University, Alexandria 21544, Egypt; r.nair@kcst.edu.kw (R.N.); s.bhat@kcst.edu.kw (S.A.B.); ishac@vt.edu (I.K.); 2 Faculty of Science, Utah State University, Logan, UT 84341, USA; 3 The Bradley Department of Electrical and Computer Engineering, Virginia Tech, Blacksburg, VA 24061, USA; 4 Center of Smart Nanotechnology and Photonics (CSNP), SmartCI Research Center of Excellence, Alexandria University, Alexandria 21544, Egypt; ahassan@ncsu.edu (A.H.H.); eman.elnabawy@smartci.alexu.edu.eg (E.E.); 5 Department of Textile Engineering, Faculty of Engineering, Alexandria University, Alexandria 21544, Egypt

**Keywords:** PVDF, nanofibers, piezoelectricity, acoustic sensors, energy harvesting

## Abstract

This paper introduces a new usage of piezoelectric poly (vinylidene fluoride) (PVDF) electrospun nanofiber (NF) membrane as a sensing unit for acoustic signals. In this work, an NF mat has been used as a transducer to convert acoustic signals into electric voltage outcomes. The detected voltage has been analyzed as a function of both frequency and amplitude of the excitation acoustic signal. Additionally, the detected AC signal can be retraced as a function of both frequency and amplitude with some wave distortion at relatively higher amplitudes and within a certain acoustic spectrum region. Meanwhile, the NFs have been characterized through piezoelectric responses, beta sheet calculations and surface morphology. This work is promising as a low-cost and innovative solution to harvest acoustic signals coming from wide resources of sound and noise.

## 1. Introduction

Alternative energy sources, such as mechanical, thermal, wind and water energies, have been extensively studied over recent decades [1,2]. These environmental resources are promising selections to be transduced into electrical engineering for both clean environment considerations and cheaper economic costs [3]. Among the different transducing mechanisms, one of the most exciting mechanisms is the piezoelectric one where mechanical energy, including vibrations, pressures, bending and tension stresses, can be converted into electrical energy [4]. This offers a promising property for a wide variety of energy harvesting applications, such as biomedical sensors [5], wearable electronics [6] and smart textiles [7]. Acoustic sensors have been widely introduced in several applications, including medical, environmental, industrial and scientific research [8,9], based on various transducing mechanisms, such as piezoelectricity, piezocapacitance, piezoresistance and piezo-optics [10,11,12,13]. Polymeric piezoelectric acoustic sensors are considered among the most distinct types of materials due to their higher sensitivity and flexibility [14]. However, most of the piezoelectric polymer processes have some drawbacks due to the need for high-voltage poling or mechanical stretching, which entail more energy consumption and higher costs. Recently, piezoelectric nanofibers (NFs) prepared through electrospinning have been strongly introduced for various applications. Many studies have indicated that electrospun NFs from piezoelectric polymers, such as poly(vinylidene fluoride) (PVDF), poly(vinylidene fluoride-co-hexafluoropropene) (PVDF-HFP) and poly(vinylidene fluoride-co-trifluoroethylene) (PVDF-TrFE), have unique piezoelectric properties without the need for stretching or poling treatments. These piezoelectric nanofibers are considered to be some of the best candidates for mechanical sensors and energy generator applications.

PVDF nanofibers were fabricated using an electrospinning technique with an applied voltage of 5 kV and a distance between the tip and the collector of 0.1 m. The solution concentration of the PVDF was 16 wt.%. The rotary speed of the collector was varied between 500, 1000, 1500 and 2000 rpm. FTIR spectra showed some peaks at 475, 510, 1275 and 1431 cm^−1^, respectively [15]. These can prove the alignment of the generated nanofibers and the formation of the polar beta phase (*β*-phase) of PVDF. Some additives to the PVDF solution, such as hydrated salt and nickel chloride hexahydrate (NiCl_2_·6H_2_O) with a concentration less than 0.5 wt.%, could increase the polar beta phase by 30% due to the ionic interaction between salt and polymer molecules [16]. Moreover, addition of carbon nanotubes (CNTs) to PVDF can improve its piezoelectric performance. Multi-walled carbon nanotubes (MWCNTs) were added by different concentrations starting from 5 to 1 wt. % with steps of 0.05 wt.%. At the beginning, the *β*-phase increased at a high rate. Then, by increasing the concentration of CNTs, the *β*-phase increased at a slow rate until it reached saturation [17,18]. Another study added MWCNTs with concentrations of 0.6, 1 and 2 wt.%. FTIR spectra showed that the infrared band at 837 and 1273 cm^−1^ became more prominent. That means an enhancement of *β*-phase with increasing concentrations of MWCNTs. On the other hand, XRD analysis showed a notable diffraction peak at 2 theta equals 19.9° [19]. This is further evidence of the formation of the *β*-phase and the positive effect of adding MWCNTs to PVDF solution for enhancing the piezoelectric effect.

Poly(vinylidene fluoride-co-hexafluoropropene) (PVDF-HFP)/poly (3,4-ethylenedioxythio-phene) (PEDOT) composite membranes with increased pore volume and high mechanical and electrical properties were introduced as piezoresistive pressure sensors [20]. The synthesized NF mat provided high sensitivity (up to 13.5 kPa^−1^) with a minimum pressure detection of ~1 Pa and stable operation over 10,000 strain cycles. Electrospun PVDF nanofibers have been reported as ultrasensitive acoustic nanogenerators. The composite membranes exhibited a high-generating output voltage of 11 V with a current density of 6 nA/cm^2^ upon repetitive mechanical stress that could light up 10 blue light-emitting diodes (LEDs) instantaneously. The development of a flexible pressure sensor based on a PVDF-TrFE nanofiber mat was also reported [21]. The developed sensor demonstrated a maximum sensitivity of 60.5 mV. N^−1^ and reliability up to a frequency of 20 Hz. A poly(vinylidene fluoride) sensor device was prepared and showed significant detection of low-frequency acoustic waves with a sensitivity as high as 266 mV Pa^−1^ [22], which can be used as an acoustic sensor that can efficiently distinguish sound waves along with noise detection.

Regarding acoustic energy harvesting, PVDF and lead zirconate titanate (PZT) piezoelectric cantilever beams were placed inside a quarter-wavelength straight-tube resonator to harvest acoustic energy at 146 and 199 Hz, respectively. A single PVDF beam produced a voltage of 0.105 V at a frequency of 146 Hz when the incident beam level was 100 dB. For the PZT beam, the maximum voltage was 1.433 V at a frequency of 199 Hz [23]. A noise from a Disk Jockey (DJ) was converted to electrical energy using a piezoelectric transducer (model 7BB-41-2). The maximum generated power was 4.96 mW [24]. A PVDF cantilever beam was fabricated and placed inside a cavity of a Helmholtz resonator. The produced power was 0.10 µW [25]. Different configurations of the Helmholz resonator were proposed to improve the acoustic energy harvesting (AEH) [26,27,28]. Many approaches to introduce AEH were illustrated, such as Helmholz resonator-based approaches, quarter-wavelength resonator-based approaches and acoustic metamaterial-based approaches [29].

However, the acoustic-to-electric energy conversion of polymeric nanofibers still needs more research investigations to be applicable. In our work, we have prepared a flexible PVDF nanofiber mat through an electrospinning method to be used for acoustic sensing applications. Detailed characterization for physical and structural properties of the pristine PVDF membrane has been investigated, such as FTIR, XRD and SEM, along with piezoelectric analysis under applied mechanical forces. In addition, the acoustic signals within variant amplitude and frequency have been converted to electrical energy using a simple hand-made setup, where the synthesized nanofiber mat is the main sensing unit. Additionally, the acoustic signals have been traced from the generated electric signal to check selective acoustic frequencies, where the nanofiber mat is effective to detect the acoustic signals with minimum distortion.

## 2. Materials and Methods

### 2.1. Materials

PVDF (Kynar, melt viscosity: 23.0–29.0) was supplied by ARKEMA (King of Prussia, PA, USA). N, N-Dimethyl Formamide (anhydrous, 98%) was purchased from LobaChemie (India). Chemicals were used without further dilution or purification.

### 2.2. Membrane Fabrication

The homogeneous PVDF (15 wt.%) solution was prepared by dissolving 3 g of PVDF powder into 20 mL of DMF and stirred overnight. The PVDF solution was then inserted into a plastic syringe tipped with a metal needle (21 gauge). The flow rate was controlled at 1 mL. h^−1^ through a NE1000 syringe pump (New Era Pump Systems, Suffolk County, NY, USA), while the high voltage of 25 kV was applied to a syringe needle using a high-voltage power supply CZE1000R (Spellman, Hauppauge, NY, USA) and collected on a rotating drum collector at 1500 rpm, which was placed 10 cm away from the needle tip. The aforementioned parameters have been proved by earlier research work done by the authors to generate the best membrane morphology with minimum beads and defects [30,31].

### 2.3. Material Characterizations

#### 2.3.1. Morphological Characterization

The morphological structure of nanofiber webs was observed using a scanning electron microscope (JEOL JSM-6010LV-SEM) with an accelerating voltage of 15 kV. The nanofiber mat was fixed onto a carbon tape and sputtered with platinum before measuring. The fiber diameter was obtained using image processing software (ImageJ, Madison, WI, USA).

#### 2.3.2. Physical Characterization

X-Ray diffraction (XRD) was measured on a diffractometer (Shimadzu Xlab 6100, Kyoto, Japan) with Cu radiation. The samples were scanned in the 2θ range of 5 to 80° and with a scanning speed of 12 (deg/min). The Fourier-transform infrared spectroscopy (FTIR) spectra were measured on a FTIR spectrophotometer (Vertex 70 FTIR, Bruker, Billerica, MA, USA) in ATR mode. Samples were scanned 120 times at a resolution of 5 cm^−1^ over a range of 4000–400 cm^−1^.

#### 2.3.3. Piezoelectric Characterization

Piezoelectric analysis of PVDF NFs was performed using a tool designed to control the applied force on PVDF nanofibers through a motorized spring. Changing the length of the spring can control the applied force on piezonanofibers. The nanofiber mat was sandwiched between two foil sheets and connected through shielded wires to a high-impedance oscilloscope (Tektronix MDO 3014). Then, peak-to-peak voltage was analyzed according to the changed applied force.

#### 2.3.4. Acoustic Sensing Analysis

**Figure 1:**
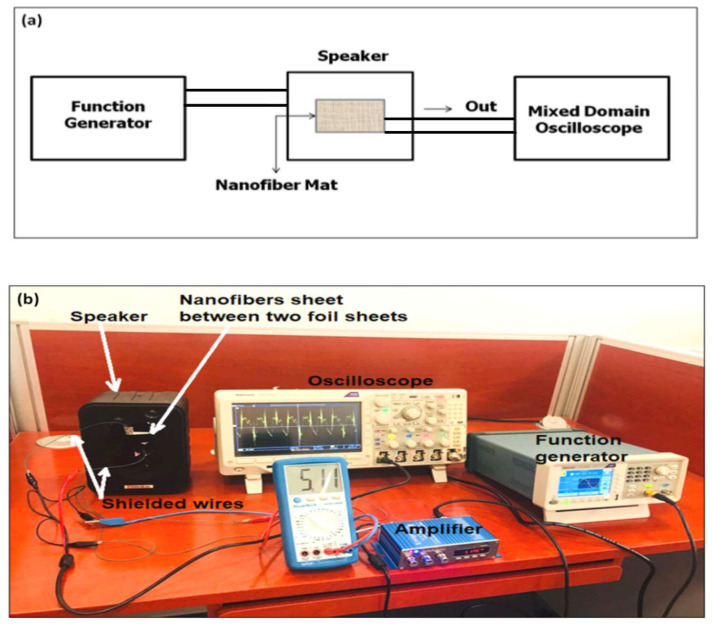
(**a**) Schematic diagram of the acoustic energy harvesting setup. (**b**) A photo of the detailed acoustic sensing setup.

Figure 1 shows a photo of the acoustic sensing setup. In this setup, a source of acoustic signals, function generator Tektronix of Model AFG1062, 60 MHz, 300 MS/s, was connected through an acoustic amplifier to a speaker. The generator controls both frequency and initial amplitude of the applied acoustic signal before being amplified. Then, the PVDF NFs mat is sandwiched between two aluminum foil parallel sheets and stacked to the speaker. The two foil sheets are connected through two shielded wires to a mixed domain oscilloscope (MDO) Tektronix 3014 with high impedance. The input voltage applied by the function generator to provide the acoustic signal was measured using a voltmeter connected to the power amplifier. The oscilloscope was used to show the retraced acoustic signal detected by the mat. Using this signal, the peak-to-peak voltage was measured, and also the frequency of the distorted received signal was recorded, to study the impact of sound waves on PVDF NFs; we used a wide range of audio signal frequencies from 100 Hz to 20 KHz. The sound waves of different frequencies at different amplitudes were applied to the NF mat. In the beginning, for each frequency, the audio signal of a relatively low amplitude was applied and the output voltage generation from the NF mat was measured using the oscilloscope connected to the mat terminals. The input sound waves were modulated by amplification using a power amplifier connected to the source (speaker) where the samples of NFs were attached. These input-applied voltages were increased in steps of 0.6 V ten times until reaching 6 V, which resulted in an unbearable sound. Each time, the output voltage generated from the NFs was measured using the oscilloscope.

## 3. Results and Discussion

### 3.1. Physical Characterizations

**Figure 2:**
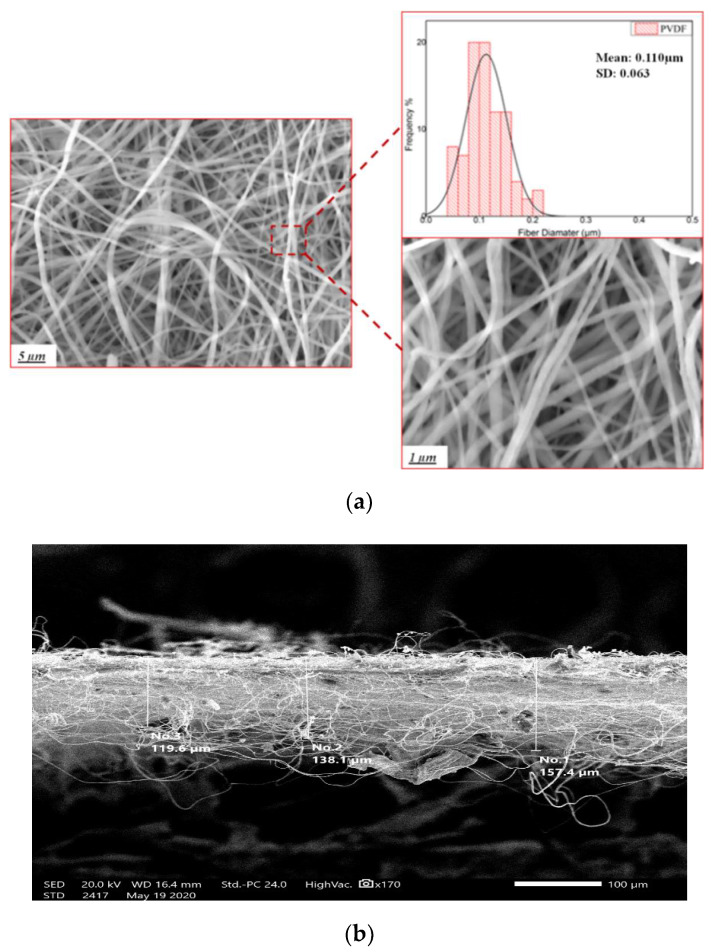
(**a**) SEM image with fiber diameter distribution for the electrospun poly (vinylidene fluoride) nanofiber (PVDF NF) mats, and (**b**) cross-section of the generated membrane.

PVDF NF morphology was examined by the FE-SEM, as shown in Figure 2. The morphological structure of the NF scaffold showed randomly distributed fibers with an average diameter of 110 ± 63 nm.

**Figure 3:**
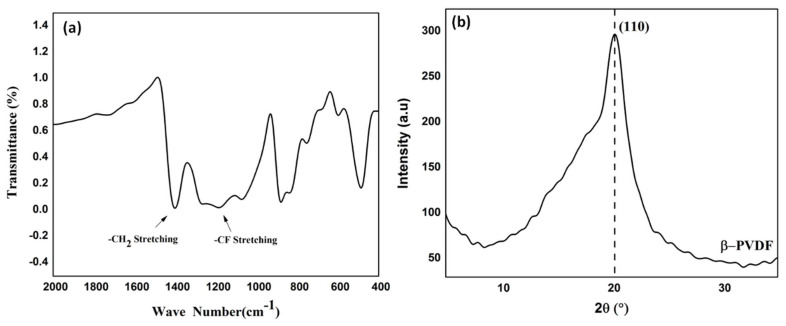
(**a**) FTIR and (**b**) XRD analyses of PVDF nanofibers.

FTIR spectroscopy and XRD were performed to analyze the degree of crystallinity and *β*-phase content for the produced PVDF NFs. Figure 3a shows the infrared spectra of the electrospun PVDF nanofibers. The graph shows characteristic bands for the C–F vibration of PVDF at 1400.2, 1191.9 and 881.4 cm^−1^ [32]. Furthermore, the *β*-absorption peak of PVDF appeared at 837.2 cm^−1^, which confirms the effect of electrospinning on enhancing the polarizability of PVDF. The amount of *β*-phase was quantitatively calculated by considering the relative absorption intensity of the *β*-phase (840 cm^−1^) and the α phase (760 cm^−1^), according to the proposed Gregorio and Cestari equation [33]:

F (*β*) = A_β_ / (1.3A_α_ + A_β_) (1)

where F (*β*) is the *β*-phase content, and A_α_ and A_β_ are the absorbance at 766 and 840 cm^−1^, respectively. By calculating the previous equation according to the obtained IR result, the *β*-phase content for the PVDF nanofiber mat was 0.75, which confirmed the high-piezoelectric response of our obtained nanofiber. Figure 3b represents the XRD pattern of the PVDF mat, the high intensity for the main characteristic peak corresponding to the PVDF *β*-phase (2θ = 20.3°), which was attributed to the crystallographic (110) plane that resulted. The *β*-phase is known to be the most polar phase of PVDF, which shows spontaneous polarization by converting the mechanical energy into electrical energy and vice versa (piezoelectric and pyroelectric activity) [34]. Thus, the formation of nanofibers by electrospinning promotes the formation of the *β*-phase, which in return enhances the piezoelectric properties.

### 3.2. Piezoelectric Characterization

**Figure 4:**
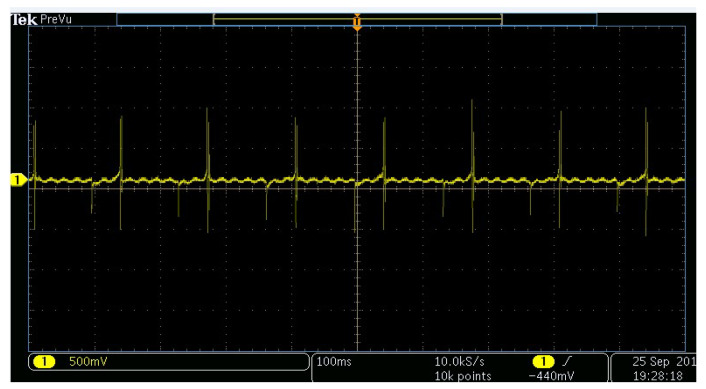
Piezoelectric analysis of the PVDF nanofiber membrane at a cyclic applied force of 5 N and at a rate of 5 Hz. The shown y-axis presents electric voltage (in volts) and the x-axis is the time (in seconds).

**Figure 5:**
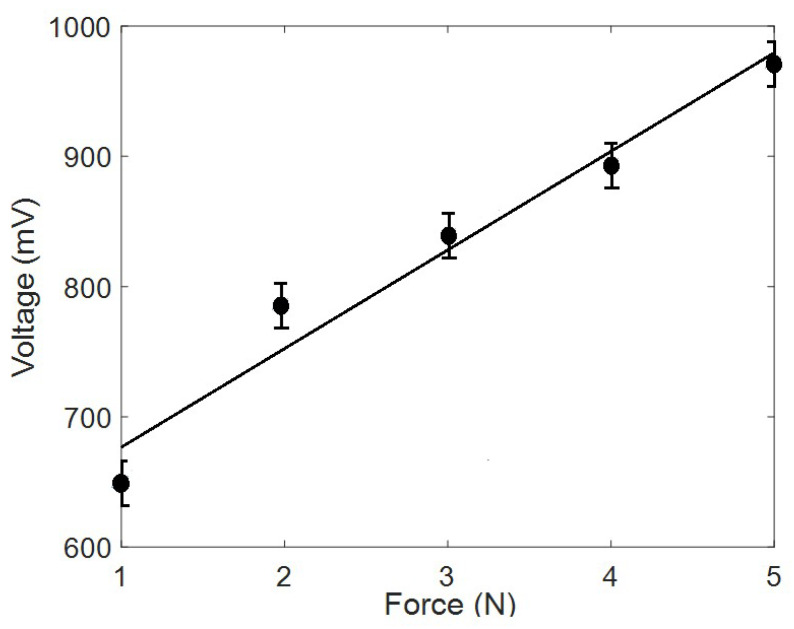
Mean peak-to-peak converted voltage through the electrospun nanofibers under the impact of applied cyclic force.

For piezoelectric analysis, different applied forces were exposed to the synthesized PVDF nanofiber mat. Figure 4 shows the generated electric voltage by applying cyclic forces on the electrospun nanofiber mat. Figure 5 shows the output peak-to-peak voltage at different applied forces. It can be noticed that our synthesized nanofibers can be sensitive to a load of a few newtons with a piezoelectric response sensitivity of 1.05 × 10^−5^ V.m/N or 0.23 mV/Pa due to formed electric dipoles inside the electrospun nanofibers.

### 3.3. Acoustic Sensing Measurements

#### 3.3.1. Impact of Acoustic Signal Frequency and Amplitude

**Figure 6:**
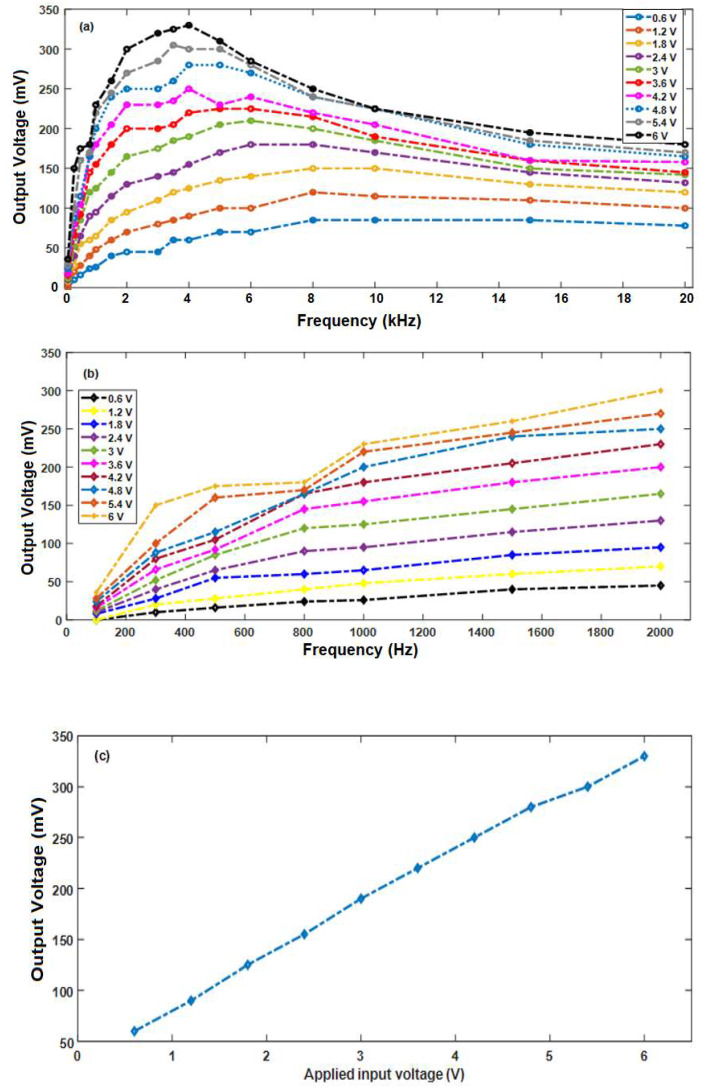
(**a**) Measured voltage versus frequency at different input applied voltages. (**b**) Measured voltage versus low frequency (up to 2 kHz) at different input applied voltages. (**c**) The output voltage versus applied input voltage at a frequency of 4 kHz.

As the frequency increased from 100 Hz to 10 kHz, the sound waves with increasing amplitude resulted in unendurable sound, but the sound waves were not audible above 10 kHz with amplitudes up to 6 V. All these cases resulted in an output voltage with some sort of distortion to the waves at certain amplitudes depending on the frequency level. Thus, the effect of audio signals of different frequencies at different amplitudes on the NF mat was studied. The output voltage versus frequency at different amplitudes obtained from the NF mat is shown in Figure 6a. For low-amplitude sound waves at input voltages from 0.6 to 1.2 V, the output voltage generated from the NF mat was found to be increasing almost linearly for low-applied voltages with increases in frequency up to 2000 Hz, as shown in Figure 6b, and then from 1 to 20 kHz, the generated output voltage was found to be almost constant. However, this behavior is found only at low-amplitude sound waves. As the input voltage increased from 1.8 to 6 V, the audio signal amplitude increased and the generated output voltage increased linearly as the frequency increased up to 4000 Hz; then, the output voltage started decreasing as the frequency increased to 20 kHz. The maximum generated voltage obtained from the NF mat was around 330 mV and was found at 4000 Hz, which may be correlated to a natural resonance response of our samples to the surface deformation at this frequency value. At this frequency, we plotted the generated output voltage as a function of the input voltage in Figure 6c, where the output voltage was linearly changing with the input voltage.

#### 3.3.2. Acoustic Signal Retracing

**Figure 7:**
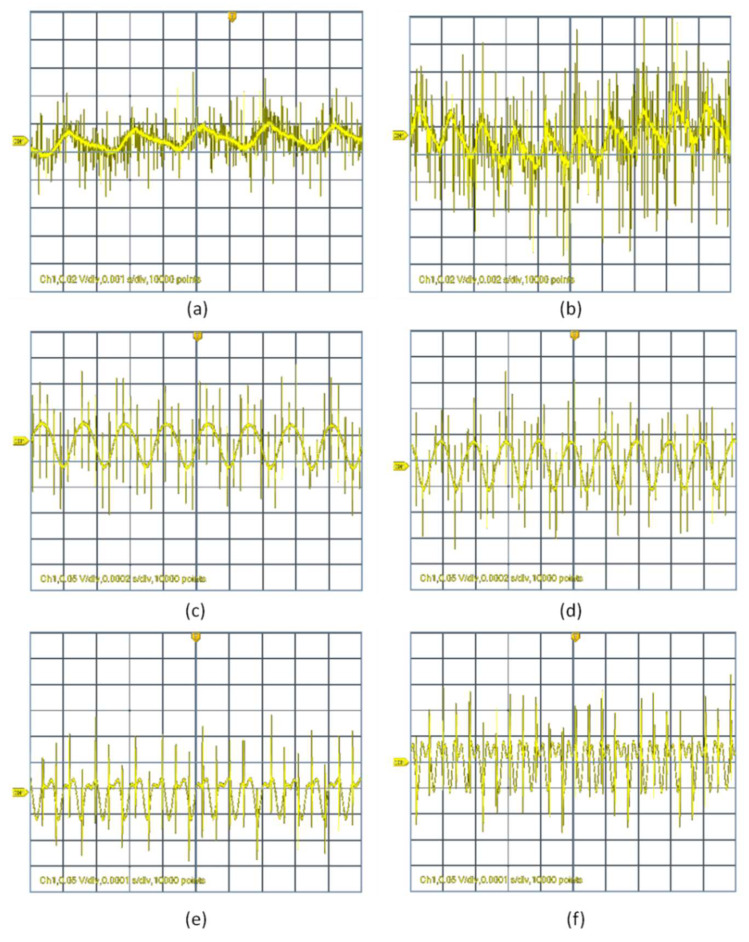
Examples of retraced acoustic wave signals at different input frequencies at an applied voltage of 1.2 volts. The used frequencies are (**a**) 300 Hz, (**b**) 500 Hz, (**c**) 4 kHz, (**d**) 5 kHz, (**e**) 15 kHz and (**f**) 20 kHz. All wave signals graphs have a y-axis of voltage (in volts) and an x-axis of time (in seconds).

**Figure 8:**
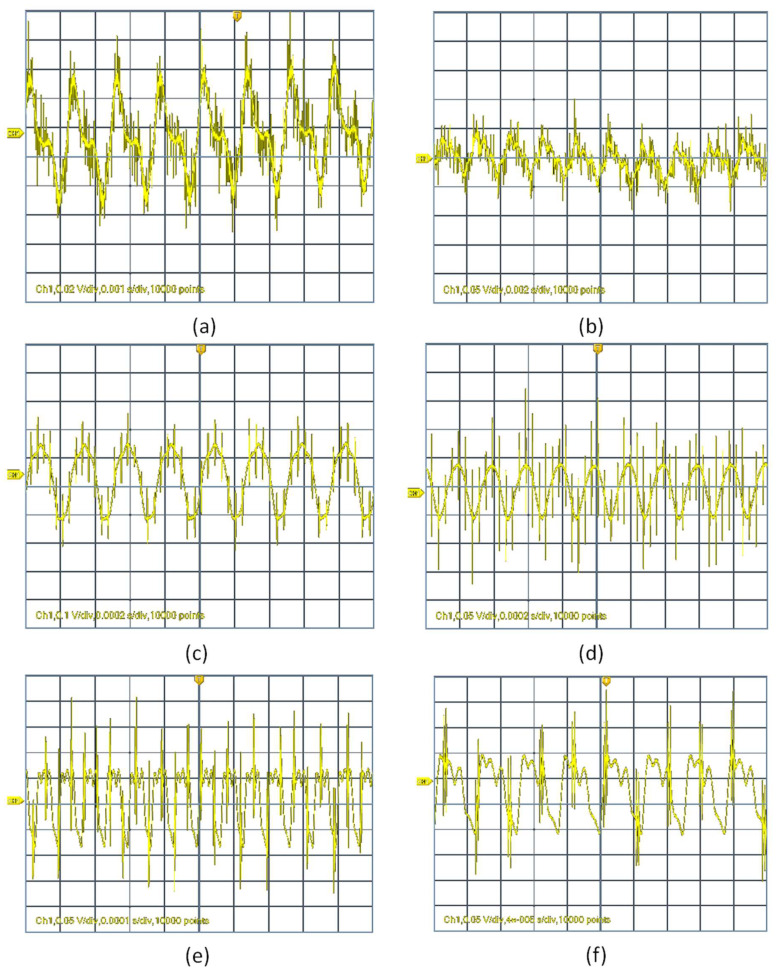
Examples of retraced acoustic wave signals at different input frequency at an applied voltage of 4.8 Volt. The used frequencies are, (**a**) 300 Hz, (**b**) 500 Hz, (**c**) 4 kHz and (**d**) 5 kHz. (**e**) represents the output signal voltage obtained for a frequency of 15 kHz and (**f**) 20 kHz. All wave signal graphs have a y-axis of voltage (in volts) and x-axis of time (in seconds).

In this section, we measure the retracing of the acoustic signal detected by the NF mat. The retracing focuses on both signal distortion and frequency detection. Examples of signal profile distortion are shown in both Figure 7 and Figure 8.

For relatively low-amplitude sound waves, the output signal was not distorted by much compared to higher amplitudes. However, at lower frequencies in the range of 100 to 800 Hz and for higher frequencies ranging from 10 to 20 kHz, the output signal was affected by distortion even at lower amplitudes. While at low-amplitude sound waves in the medium range, frequencies from 1 to 8 kHz had no distortion compared to other frequencies. Here in the case of medium range frequency, the output signals were affected by distortion only at the highest amplitude level, but the distortion level was very low compared to all other frequencies involved in our studies.

Both Figure 7 and Figure 8 clearly depict the distortion level of output signals for different input frequencies (300 Hz, 500 Hz, 4 kHz, 5 kHz, 15 kHz and 20 kHz) at an input applied voltage of 1.2 and 4.8 V, respectively. As the input applied voltage increased to 4.8 V, the distortion rate increased a lot at low and high frequencies except in the medium range frequency, which is 4 kHz, as shown in the figure. In the case of low frequencies (100 to 800 Hz) and for high frequencies (10 to 20 kHz), the distortion level was very high and the distortion level was found to increase linearly with increases in input sound wave amplitudes. Therefore, based on studies in a range of frequencies with different amplitudes, the NF mat had an optimum performance in the range of 1 to 8 kHz with maximum output voltage without much distortion.

For frequency retracing, Table 1 shows the detected frequencies compared to the input acoustic signal frequency with the calculated percentage error. It can be shown that our synthesized NF mat shows a relatively low-retracing frequency error for the whole acoustic frequency spectrum. The maximum absolute error in frequency detection or retracing was found to be 1.6%.

## 4. Conclusions

In this paper, we have presented a new application of PVDF NFs as a sensor for acoustic signals. The piezoelectric PVDF NF membrane, synthesized by electrospinning, has been used as a target for acoustic excitation waves at different amplitudes and frequencies. The synthesized NFs transduced the acoustic signals into electric potential based on their piezoelectric properties. In addition, the fibers can retrace the input acoustic signals with less distortion at a certain acoustic spectrum region with relatively low-amplitude acoustic excitations. This work is promising for harvesting acoustic signals and noise and transducing them into electrical energy.

## Figures and Tables

**Table 1:** Measured frequency of the generated signals from the PVDF NF mat compared to input acoustic signals with percentage error calculations.

Input Frequency (Hz)	Output Frequency (Hz)	Absolute Percentage Error (%)
800	787	1.6
1000	990	1
3000	3012	0.4
6000	5924	1.2
8000	8012	0.15
10,000	9920	0.8
15,000	15,197	1.3
20,000	19,960	0.2
